# Ion-Complex Microcrystal Formulation Provides Sustained Delivery of a Multimodal Kinase Inhibitor from the Subconjunctival Space for Protection of Retinal Ganglion Cells

**DOI:** 10.3390/pharmaceutics13050647

**Published:** 2021-05-01

**Authors:** Henry T. Hsueh, Yoo-Chun Kim, Ian Pitha, Matthew D. Shin, Cynthia A. Berlinicke, Renee Ti Chou, Elizabeth Kimball, Julie Schaub, Sarah Quillen, Kirby T. Leo, Hyounkoo Han, Amy Xiao, Youngwook Kim, Matthew Appell, Usha Rai, HyeYoung Kwon, Patricia Kolodziejski, Laolu Ogunnaike, Nicole M. Anders, Avelina Hemingway, Joan L. Jefferys, Abhijit A. Date, Charles Eberhart, Thomas V. Johnson, Harry A. Quigley, Donald J. Zack, Justin Hanes, Laura M. Ensign

**Affiliations:** 1 Center for Nanomedicine at the Wilmer Eye Institute, Johns Hopkins University School of Medicine, Baltimore, MD 21231, USA; thsueh2@jhu.edu (H.T.H.); uchunkim@gmail.com (Y.-C.K.); ipitha1@jhmi.edu (I.P.); mdshin3@gmail.com (M.D.S.); kleo1@alumni.jh.edu (K.T.L.); zingouki@gmail.com (H.H.); axiao4@jhu.edu (A.X.); youngwook.k@gmail.com (Y.K.); mappell1@jhmi.edu (M.A.); urai2@jhmi.edu (U.R.); hyjkwon@umich.edu (H.K.); pkolodz2@jhu.edu (P.K.); ogunnaike1998@gmail.com (L.O.); dateabhi@hawaii.edu (A.A.D.); ceberha@jhmi.edu (C.E.); johnson@jhmi.edu (T.V.J.); hquigley@jhmi.edu (H.A.Q.); donzack@gmail.com (D.J.Z.); hanes@jhmi.edu (J.H.); 2 Department of Chemical & Biomolecular Engineering, Johns Hopkins University, Baltimore, MD 21218, USA; 3 Department of Ophthalmology, Wilmer Eye Institute, Johns Hopkins University School of Medicine, Baltimore, MD 21287, USA; cdoughe1@jhmi.edu (C.A.B.); fcone1@jhmi.edu (E.K.); jschaub6@jhmi.edu (J.S.); squille1@jhmi.edu (S.Q.); jjeffer4@jhmi.edu (J.L.J.); 4 Department of Computational Biology, Bioinformatics, and Genomics, Center for Bioinformatics and Computational Biology, University of Maryland, College Park, MD 20742, USA; rchou@umd.edu; 5 Department of Biomedical Engineering, Johns Hopkins University, Baltimore, MD 21218, USA; 6 Department of Biophysics, Johns Hopkins University, Baltimore, MD 21218, USA; 7 Department of Pharmacology and Molecular Sciences, Johns Hopkins University, Baltimore, MD 21287, USA; 8 The Sidney Kimmel Comprehensive Cancer Center, Johns Hopkins University, Baltimore, MD 21287, USA; nander16@jhmi.edu (N.M.A.); aheming1@jhmi.edu (A.H.); 9 Departments of Neuroscience, Molecular Biology and Genetics, and Genetic Medicine, Johns Hopkins University School of Medicine, Baltimore, MD 21287, USA; 10 Departments Gynecology and Obstetrics and Infectious Diseases, Johns Hopkins University, Baltimore, MD 21287, USA

**Keywords:** neuroprotection, tyrosine kinase inhibitor, ocular drug delivery, pamoic acid, sunitinib, glaucoma

## Abstract

Glaucoma is the leading cause of irreversible blindness worldwide. Elevated intraocular pressure (IOP) is one of the major risk factors for glaucoma onset and progression, and available pharmaceutical interventions are exclusively targeted at IOP lowering. However, degeneration of retinal ganglion cells (RGCs) may continue to progress despite extensive lowering of IOP. A complementary strategy to IOP reduction is the use of neuroprotective agents that interrupt the process of cell death by mechanisms independent of IOP. Here, we describe an ion complexation approach for formulating microcrystals containing ~50% loading of a protein kinase inhibitor, sunitinib, to enhance survival of RGCs with subconjunctival injection. A single subconjunctival injection of sunitinib-pamoate complex (SPC) microcrystals provided 20 weeks of sustained retina drug levels, leading to neuroprotection in a rat model of optic nerve injury. Furthermore, subconjunctival injection of SPC microcrystals also led to therapeutic effects in a rat model of corneal neovascularization. Importantly, therapeutically relevant retina drug concentrations were achieved with subconjunctival injection of SPC microcrystals in pigs. For a chronic disease such as glaucoma, a formulation that provides sustained therapeutic effects to complement IOP lowering therapies could provide improved disease management and promote patient quality of life.

## 1. Introduction

Glaucoma is the leading cause of irreversible blindness, impacting 80 million people worldwide [1,2]. Due to the rapid increase in the aging population, the number is expected to rise to 111 million by 2040 [3]. Glaucoma is a neurodegenerative disease that causes irreversible vision loss due to the death of retinal ganglion cells (RGCs). All of the current standard-of-care treatments for glaucoma are aimed at lowering intraocular pressure (IOP). However, maintaining lower pressure can be challenging in some patients, and in others, even if significant pressure lowering is achieved, RGC loss continues [4]. We previously developed a primary cell-based method to perform high-throughput, functional genomic screening and we and others identified dual leucine zipper kinase (DLK; MAP3K12) and leucine zipper kinase (LZK; MAP3K13) as key mediators of RGC cell death in response in axonal injury [5,6,7]. Furthermore, we have identified compounds with activity against DLK and LZK that potently promote survival of mouse and human RGCs, including the FDA-approved multi-kinase inhibitor sunitinib [6]. Development of sunitinib into a formulation for effective, sustained intraocular delivery as a complement to IOP-lowering strategies could improve glaucoma treatment and management [8,9,10,11].

Patient adherence, which is critical for treating chronic diseases like glaucoma, is a clinical challenge because administration difficulties and side effects associated with glaucoma eye drop treatments result in high levels of non-compliance [12]. Typically, only 40–60% of patients adhere to glaucoma drop therapy regimens, even in cases where the patients know they are being monitored and were provided free medication [13,14,15]. Furthermore, achieving effective drug delivery to the posterior segment with an eye drop is challenging [16,17,18,19]. The most common approach for achieving drug delivery to the posterior segment is intravitreal (IVT) injection, which is employed every 6–8 weeks to deliver biologics for treatment of wet age-related macular degeneration [20,21]. However, IVT injection is relatively invasive, and is associated with rare, but severe ocular complications [22,23]. For a disease that may require treatment for decades, a less invasive injection approach is preferred. Furthermore, longer times between injections would be preferred by glaucoma patients, particularly since it may take years to notice deterioration of vision.

Here, we describe an approach for formulating sunitinib for prolonged therapeutic effects with subconjunctival injection. We previously observed that the water-soluble salt form of drugs, including sunitinib malate, was preferred for achieving intraocular drug penetration upon subconjunctival injection [24,25]. However, high water solubility presents a challenge to achieving sustained drug release using conventional encapsulation approaches. We screened water-soluble counterion salts for the ability to reversibly form an insoluble complex with sunitinib, which would lower water solubility to facilitate formulation while allow for the release of water-soluble drug. Then, rather than load the insoluble complex into a polymeric particle [26], we aimed to formulate crystalline drug complex microcrystals. One potential advantage of formulating microcrystals rather than polymeric microparticles is higher overall drug loading. Herein, we aimed to develop a less invasive injectable system for sustained neuroprotection in glaucoma, and to demonstrate that therapeutically relevant drug concentrations could be obtained in large animals. We observed that a single subconjunctival injection of sunitinib-pamoate complex (SPC) microcrystals provided at least 20 weeks of RGC protection in a rat optic nerve crush model of RGC injury. Importantly, we also confirmed that therapeutically relevant sunitinib concentrations were achievable upon subconjunctival injection of SPC microcrystals in the larger eyes of pigs [27].

## 2. Materials and Methods

### 2.1. Material Sources

Sunitinib malate was purchased from LC Laboratories (Woburn, MA, USA). Pamoic acid disodium was purchased from Tokyo Chemical Industry (Chuo-ku, Tokyo, Japan). *N*-desethyl sunitinib and sunitinib-d10 were purchased from Toronto Research Chemical (North York, ON, Canada). Phosphate buffered saline 1X (PBS), neurobasal media, calcein AM (acetomethoxy derivate of calcein), ethidium homodimer, Hoechst 33342, B-27, N-2, l-glutamine, penicillin/streptomycin, goat anti-rabbit IgG (H+L) Alexa Fluor 568, 4’,6-diamidino-2-phenylindole dihydrochloride (DAPI), 2-mercaptoethanol, bovine serum albumin and fluoromount-G were purchased from Thermo Fisher Scientific (Waltham, MA, USA). CellTiter-Glo luminescent cell viability assay was purchased from Promega (Madison, WI, USA). Goat anti-mouse IgG H&L Alexa Fluor^®^ 647 was purchased from Abcam (Cambridge, MA, USA). High-performance liquid chromatography (HPLC) grade acetonitrile and water were purchased from Fisher Scientific (Hampton, NH, USA). Nylon net filters (20 μm) were purchased from Millipore (St. Louis, MO, USA). Mesh strainers (35 μm) were purchased from Corning (Corning, NY, USA). Deuterated dimethyl sulfoxide D-6 was purchased from Cambridge Isotope Laboratories (Tewksbury, MA, USA). Polysorbate 80 (PS80), Lutrol F127, trifluoroacetic acid, Triton X-100, diethyl ether, acetone, and biological grade ethanol were purchased from Sigma Aldrich (St. Louis, MO, USA). Methylcellulose (400 cP) was purchased from Spectrum Chemical (New Brunswick, NJ, USA). Reverse-action forceps were purchased from World Precision Instruments (Sarasota, FL, USA). Rabbit-anti γ-synuclein was purchased from GeneTex (Irvine, CA, USA). Mouse-anti β-III tubulin was purchased from BioLegend (San Diego, CA, USA). Nylon sutures (10-0) were purchased from Ethicon (Cincinnati, OH, USA). Neomycin, polymyxin B, and bacitracin zinc ophthalmic ointment was purchased from Akorn (Lake Forest, IL, USA). ISOTON^®^ Diluent was purchased from Beckman Coulter (Indianapolis, IN, USA).

### 2.2. SPC Synthesis and Characterization

Negatively charged hydrophobic salts, including pamoic acid disodium salt, linoleic acid sodium salt, sodium stearate, and sodium decanoate, were screened for complexation with sunitinib malate. The hydrophobic salts and sunitinib malate were each dissolved separately at 0.5 mg/mL in 15 mL of ultra-pure, endotoxin-free water. Various weight ratios (1:1, 2:1, 5:1, 8:1, 10:1, 20:1) of pamoic acid disodium salt to sunitinib and different temperature settings (freezer, refrigerator, room temperature, and water bath at 37 °C and 54 °C) were screened for complexation efficiency after incubation for 24 h by visually observing the size of the pellet after centrifugation at 4000× *g* for 30 min. To make the sunitinib-pamoate complex (SPC) for particle formulation, sunitinib malate (60 mg) and pamoic acid disodium salt (600 mg) were dissolved separately in 15 mL of ultra-pure, endotoxin-free water and were further mixed and by brief vortexing at room temperature for 5 s. The mixture was kept in the dark for 2 h, then collected by centrifugation at 4000× *g* for 30 min, and lyophilized (Labconco) for further characterization.

The SPC drug loading study was determined by measuring the sunitinib concentration from the SPC dissolved with DMSO. The SPC were weighed and dissolved with 1 mL of the DMSO and further diluted 200× with the DMSO. The sunitinib concentration were measured at λ_em_ = 540 and further used for calculating the drug loading percentage based on the original weighed SPC (*n* = 6). Nuclear magnetic resonance (NMR) was also used to determine the ratio of sunitinib to pamoate in SPC. Sunitinib malate, pamoic acid, and SPC were separately dissolved in deuterated dimethyl sulfoxide. ^1^H NMR spectra were recorded on a Bruker spectrometer (500 MHz), and the data were processed using iNMR software. ^1^H chemical shifts were reported in ppm (δ) and the DMSO peak was used as an internal standard. The molar ratio of sunitinib malate and pamoic acid disodium in the SPC complex was determined by the taking the ratio of sunitinib peak at δ = 1.2 to pamoic acid peak δ = 4.8.

To measure solubility, 5 mg of sunitinib malate or SPC complex was placed in microcentrifuge tubes with 1 mL of PBS. The samples were then placed on an orbital shaker (150 rpm) in an incubator at 37 °C. After 7 days, samples were collected and centrifuged at 17,000× *g* for 30 min. The supernatant was collected, and concentrations were measured using HPLC (Shimadzu Prominence LC-20). Supernatant samples were mixed 1:10 with acetonitrile containing 0.1% trifluoroacetic acid. Acetonitrile with water was used as a mobile phase at a ratio of 55:45. Samples were eluted isocratically at a flow rate of 1 mL/min through a C18-reversed phase column at 40 °C. UV absorbance was monitored at 431 nm.

### 2.3. SPC Microcrystal Formulation and Characterization

Different volume ratios (0%, 20%, 40%, 60%, 80%, 100%) of acetone, ethanol, and diethyl ether in water were screened for dissolving SPC for recrystallization. The solutions of SPC in ethanol:water (60 to 100%) were then stirred at different temperatures (refrigerator, room temperature, water bath at 37 °C) to evaporate solvent and facilitate crystallization. To make the final SPC microcrystal formulation, SPC solids (78 mg ± 5.2 mg) were dissolved by adding 2.7 mL of 70% ethanol/water and vortexing vigorously for 5 min. The solution was then stirred in a fume hood at 60 rpm for 2 h to evaporate solvent and facilitate crystallization. The resulting SPC microcrystals were then collected by pouring through a 20 µm net filter, followed by three times of washing each with 50%, 20%, and 0% of ethanol in water. Various surface stabilizers were then added to improve crystal stability. Suspending the crystals in PS80 (0.01–1%), Pluronic F68 (0.05–1%), Pluronic F127 (0.005–1%), and Pluronic L61 (0.05–0.1%) all resulted in either an increase in the heterogeneity of the crystal size or crystal dissolution within 24 h after addition (confirmed by visual observation under a light microscope). In contrast, the size of the microcrystals was stable at room temperature without a stabilizer when kept at a concentration of at least 5 mg/mL, and thus a stabilizer was not incorporated in the final formulation.

Drug loading (*n* = 6) was calculated by dissolving a known mass of lyophilized crystals and determining the sunitinib content by HPLC (details above), and the yield was calculated as a percentage of the starting amount of sunitinib malate (*n* = 3). Crystal size and morphology was characterized both by Coulter counter (Multisizer 4e, Beckman Coulter Life Sciences) and light microscopy. To reduce microcrystal aggregation observed when adding to the Coulter ISOTON^®^ Diluent, 1% (*w*/*w*) F127 was added immediately prior to size measurements. SPC microcrystals (200 µL at 5 mg/mL sunitinib in 1% F127) were added to 100 mL of ISOTON diluent. The particle size was quantified until the total particle count reached 100,000 particles. To visualize microcrystal morphology, 5 µL of solution (40 mg/mL in 1% (*w*/*w*) F127 to prevent aggregation upon drying) was placed on a glass slide with a coverslip and imaged with a Nikon ECLIPSE Ni-E bright field microscope (Melville, NY USA) at 40×. At least 3 images were taken, and *n* = 10 crystals were measured per sample (*n* = 3 samples). ImageJ - Fiji software (MacOS, Java 8 with 2017 May 30 Version) was used to measure the size of the SPC crystals. For the crystal stability test, 200 µL of 5 mg/mL of SPC microcrystals in water were aliquoted into 1.5 mL Eppendorf tubes and kept at room temperature in the dark for up to 180 days. The crystal size was measured at 0, 21, 35, 120, and 180 days by Coulter counter.

A powder X-ray diffractometer (XRD, X’Pert PRO MPD) was used to characterize SPC microcrystal crystallinity. SPC microcrystals suspended in water were lyophilized (Labconco) for 1 week to obtain powdered SPC. The SPC powders were packed to the sample holder with a flat surface and located against the reference plane in the center of the goniometer. The start and end angles were set to 5° and 100°, with a step size of 0.02° and time per step set as 10 s. The tension was set to 45 kV and the current to 40 mA. Background subtraction and smoothing were applied to the dataset for qualitative analysis. The intensity of the X-ray diffraction patterns was recorded as a function of the angle of the detector (2θ).

### 2.4. Retinal Ganglion Cell (RGC) Viability Assay

Mouse RGCs were purified as described previously [5], plated on a poly-d-lysine/laminin coated 384-well culture dish at 4000 cells per well and cultured in Neurobasal media supplemented with B-27, N-2, l-glutamine, and penicillin/streptomycin. Sunitinib malate (1 µM) was dissolved in dimethyl sulfoxide (DMSO) and added at the same time cells were plated (250 nL into 50 µL of media) to promote RGC survival. Then, either PBS or different volumes of sodium pamoate dissolved in DMSO was added at the concentrations indicated (0.1, 1, 10 mg/mL) to determine whether there was any effect on RGC survival. A PBS control was included for each pamoic acid concentration to account for dilutional effects. After cells were cultured for 72 h at 37 °C, cell viability was measured by CellTiter-Glo (Promega) luminescence according to the manufacturer’s protocol.

### 2.5. Animal Welfare Statement

All procedures were approved by the Johns Hopkins Animal Use and Care Committee under protocol numbers RA19M120 (1 January 2019), RB18M280 (1 January 2018) and SW18M268 (1 January 2018). All animals were handled and treated in accordance with the Association for Research in Vision and Ophthalmology Statement for Use of Animals in Ophthalmic and Vision Research. For rats and rabbits, sex was not specified when ordering any species, and approximately equal amounts of male and female animals were used as provided. For the pigs, *n* = 2 male and *n* = 2 female animals were used. Brown Norway and Wistar rats, and New Zealand White rabbits were obtained from Charles River (Frederick, MD, USA). Yorkshire pigs were obtained from Archer Farm (Darlington, MD, USA). All animals received general anesthesia (either intraperitoneal injection of ketamine/xylazine or isoflurane inhalation where noted) in addition to topical anesthesia (0.5% proparacaine hydrochloride) prior to ocular injections and procedures. Animals were anesthetized prior to euthanasia.

### 2.6. Pharmacokinetics

#### 2.6.1. Effect of Injection Volume and Dose on Drug Concentration in the Posterior Segment

Wistar rats (6–8 weeks old) and New Zealand White rabbits (2–3 kg) were used. Animals received a unilateral subconjunctival injection of SPC microcrystals using a 27-gauge needle. To look at the effect of dose in rats, four different doses of SPC microcrystals (5, 50, 100, 200 µg) were suspended in carrier fluid containing 0.04% (*w*/*v*) PS80 with 0.5% (*w*/*v*) methylcellulose in saline and injected in 5 µL volume. At 7 days after the injection, the eyes were enucleated and separated into the retina and choroid/retinal pigmented epithelium (Ch/RPE) for analysis of sunitinib and its major metabolite, *N*-desethyl sunitinib. We previously found that sunitinib and *N*-desethyl sunitinib had similar neuroprotective activity in both primary mouse RGCs and human stem cell-derived RGCs [28], and thus drug concentrations were combined in pharmacokinetic analyses. To look at the effect of injection volume, SPC microcrystals were suspended in carrier fluid containing 0.04% (*w*/*v*) PS80 with 0.5% (*w*/*v*) methylcellulose in saline for the 5 µL injections in rats and rabbits. For the higher injection volumes (100 µL in rats and 500 µL in rabbits), the overall material amounts (200 µg sunitinib, 2 µg PS80, 25 µg methylcellulose) in saline were matched. At 7 days after the injection, the eyes were enucleated and separated into the retina and choroid/retinal pigmented epithelium (Ch/RPE) for analysis of sunitinib and *N*-desethyl sunitinib content.

#### 2.6.2. Drug Concentrations in the Posterior Segment 

Brown Norway rats (6–8 weeks old) and juvenile Yorkshire pigs (20–30 kg) were used. Rats received a 5 µL unilateral subconjunctival injection of SPC microcrystals (200 µg sunitinib, 0.04% PS80, 0.5% methylcellulose) using a 27-gauge needle. At 1, 3, 5, and 20 weeks after the injection, the eyes were enucleated and separated into the retina and Ch/RPE for analysis of sunitinib and *N*-desethyl sunitinib content. Drug levels were combined and reported. Pigs received a 5 µL (200 µg sunitinib, 0.04% PS80, 0.5% methylcellulose) or 50 µL (2 mg sunitinib, 0.04% PS80, 0.5% methylcellulose) unilateral subconjunctival injection of SPC microcrystals using a 27-gauge needle. After 7 days, the animals were sacrificed, and the retina and Ch/RPE were collected for analysis for sunitinib malate and *N*-desethyl sunitinib content. Drug levels were combined and reported.

#### 2.6.3. Effect of Injection Volume on Drug Concentration in the Cornea

Rats received a unilateral anterior subconjunctival injection of SPC microcrystals (200 µg sunitinib) using a 27-gauge needle. For the 5 μL injections, SPC microcrystals were suspended in carrier fluid containing 0.04% (*w*/*v*) PS80 with 0.5% (*w*/*v*) methylcellulose in saline. For the 100 μL injections in rats, the dose was matched (200 µg sunitinib, 2 µg PS80, 25 µg methylcellulose). At 7 days after the injection, the eyes were enucleated, and the cornea was excised for analysis of sunitinib and *N*-desethyl sunitinib content. As sunitinib and *N*-desethyl sunitinib have been shown to have similar potency in inhibiting VEGF [29], drug levels were combined and reported.

### 2.7. Measurement of Sunitinib and N-Desethyl Sunitinib in Ocular Tissues

All the obtained tissues were collected in pre-weighed tubes and stored in −80 °C freezer until analysis. Liquid chromatography-tandem mass spectrometry (LC-MS/MS) was used to measure drug concentrations as previously described [27]. Briefly, tissue samples were homogenized in 200–500 µL 1X PBS using Next Advance Bullet Blender before drug extraction. Sunitinib was extracted from 50 µL of tissue homogenates with 0.150 mL of acetonitrile containing 2.5 ng/mL of the internal standard, sunitinib-d10. After centrifugation, the top layer was then transferred autosampler vial for LC-MS/MS analysis. All ocular tissue samples were analyzed using a 1X PBS standard curve. The separation was achieved with a Waters Cortecs C18 (2.1 × 50 mm, 2.7 μm) column at room temperature using a gradient. Water containing 0.1% formic acid was mobile phase A, and acetonitrile containing 0.1% formic acid was mobile phase B. Mobile phase B was held at 10% for 0.5 min and increased to 100% over 0.5 min. Mobile phase B was held at 100% for 1 min, and then returned back to 10% and allowed to equilibrate for 1 min. The total run time was 3 min with a flow rate of 0.3 mL/min. The column effluent was monitored using a Sciex triple quadrupole 5500 mass-spectrometric detector (Sciex, Foster City, CA, USA) using electrospray ionization operating in positive mode. The spectrometer was programmed to monitor the following MRM transition 399.1 → 283.2 for sunitinib, 371.2 → 283.2 for *N*-desethyl sunitinib and 409.1 → 283.2 for the internal standard (sunitinib-d10). Calibration curves were computed using the area ratio peak of the analysis to the internal standard by using a quadratic equation with a 1/x^2^ weighting function over the range of 0.1–100 ng/mL for sunitinib with dilutions of up to 1:100 (*v*:*v*).

### 2.8. Rat Optic Nerve Crush Model

Brown Norway rats (6–8 weeks old) underwent optic nerve crush and sacrifice 2 weeks later. Treated rats received a unilateral subconjunctival injection of SPC microcrystals (200 µg/5 µL sunitinib, 0.04% PS80, 0.5% methylcellulose) using a 27-gauge needle at either 3, 8, or 20 weeks prior to sacrifice. Control animals were given a unilateral subconjunctival injection of 5 µL of vehicle (0.04% PS80, 0.5% methylcellulose) at the time of the crush procedure. To ensure that there was no effect of the age of the rat at the time of the crush procedure on RGC survival, control groups were included at both the 3- and 20-week timepoints. For the crush procedure, rats were anesthetized with ketamine/xylazine and received topical anesthesia with 0.5% proparacaine hydrochloride. The temporal conjunctiva of the left eye was grasped with 0.12 mm toothed forceps and incised parallel to the limbus with sharp iris scissors. Dissection was performed using two pairs of curved blunt-tipped forceps, and the orbital fat and soft tissue were retracted to expose the orbital portion of the optic nerve. The optic nerve was crushed at a position 1.5–2 mm posterior to the globe using reverse-action forceps for 20 s. The orbital soft tissue was then repositioned over the nerve and the conjunctiva was left to close by secondary intention. After the procedure, topical bacitracin-neomycin-polymyxin ophthalmic ointment was applied to both eyes to prevent infection. Animals were sacrificed for tissue collection 14 days after the crush procedure.

### 2.9. Rat Model of Laser-Induced Intraocular Pressure (IOP) Elevation

Translimbal laser treatment in rats induces an increase in IOP and loss of RGCs judged by both axonal and cell body loss [30]. Rats were given unilateral, subconjunctival injections of 200 µg SPC microcrystals (200 µg/5 µL sunitinib, 0.04% PS80, 0.5% methylcellulose) or vehicle only (5 µL containing 0.04% PS80, 0.5% methylcellulose, Sham) using a 27-gauge needle (day-7). The contralateral eye was used as a control. Seven days after microcrystal injection, a 532-nm laser was used to induce ocular hypertension by scarring the trabecular meshwork (day 0). Animals received general anesthesia with ketamine/xylazine, and a drop of 0.5% proparacaine hydrochloride was used to anesthetize the eye. An ophthalmic surgeon who was masked to treatment group applied the laser at 45 to 55 spots at 50 μm size, 0.6-W power, and 0.6-s duration. Topical 5% erythromycin ointment was applied at the end of each procedure. IOP was measured under sedation by isoflurane inhalation on days 2, 5, 9, 14, 28, 35, and 40 following microcrystal injection using a TonoLab tonometer (iCare, Vantaa, Finland) calibrated for the rat eye. Topical anesthesia was not used for IOP measurement. The tonometer was used according to the manufacturer’s instructions with the magnetic probe in a horizontal position. Three measurements, each consisting of the mean of 6 recordings, were taken for each eye. Animals were sacrificed on day 42–45 after laser for further analyses.

### 2.10. Axial Length and Width Measurement

Measurement of axial length and width was described previously [31]. Briefly, rats were sacrificed, followed by intracardiac perfusion with 4% paraformaldehyde. The eyes were enucleated, and a needle connected to a fluid-filled reservoir was placed in the eye to set the IOP to 15 mm Hg. Digital calipers (Instant-Read-out Precision Digital Caliper; Electron Microscopy Sciences, Hatfield, PA, USA) were used to measure the length (center of the cornea to a position just temporal to the optic nerve) and width (largest dimension at the equator, midway between the cornea and optic nerve).

### 2.11. Optic Nerve Axon Counting

Axonal loss was quantified using a published method [30]. Rats were sacrificed and transcardially perfused with 4% paraformaldehyde in phosphate buffer. The eyes were enucleated with the optic nerve attached and fixed for an additional 1 h in 4% paraformaldehyde prior to optic nerve dissection. A cross section of the optic nerve was removed 1.5 mm posterior to the globe and postfixed in 1% osmium tetroxide in phosphate buffer. Nerves were processed into epoxy resin, sectioned at a thickness of 1 μm, and stained with 1% toluidine blue. The area of the optic nerve cross section was measured by outlining the outer border at 10× magnification (Sensys digital camera and Metamorph software; Universal Imaging Corp., West Chester, PA, USA). Images were captured at 100× from 10 randomly spaced nerve regions using a phase-contrast objective to measure the density and fiber diameter distributions. A technician masked to treatment group viewed the images to eliminate nonneural objects, and to measure the size of each axon internal to its myelin sheath (minimum diameter) and the density of axons per square millimeter. The mean density was multiplied by nerve area to yield fiber number for each nerve. The counting process was performed by observers masked to the protocol used in each nerve. The total axon number in the glaucomatous eye was compared with pooled, control fellow eyes to yield a percentage loss value.

### 2.12. Retinal Ganglion Cell Staining, Imaging, and Counting

For the crush studies, rats were sacrificed and the eyes harvested with the optic nerve attached. The retinas were removed, incised for flat mounting, and post-fixed for 1 h. For the laser studies, harvested eyes were post-fixed for 1 h (as described above), and the retinas were removed, incised, flat mounted, and fixed for an additional 24 h. Retinal tissue was then stored in phosphate buffer prior to staining. Retinas were then washed with 0.5% Triton-100 in PBS for 30 min, and incubated for 3 days at 4 °C in a solution containing rabbit-anti γ-synuclein (1:250 dilution), mouse-anti βIII tubulin (1:500 dilution), 1% Triton X-100, and 1% bovine serum albumin in PBS. The retinas were then washed three times with 0.5% Triton-100 in PBS, and further incubated overnight at 4 °C in a solution containing goat anti-mouse IgG H&L Alexa Fluor 647 (1:1000 dilution) and goat anti-rabbit IgG H&L Alexa Fluor 568 (1:1000 dilution) secondary antibodies in 1% Triton X-100, and 1% bovine serum albumin in PBS, or the same solution containing goat anti-rabbit IgG H&L secondary antibody Alexa Fluor 555 (1:1000 dilution), 1% Triton X-100, and 1% bovine serum albumin in PBS. The retinas were washed three times and incubated overnight in DAPI diluted 1:1000 in PBS. The stained retinal tissue was then mounted on a slide using Fluoromount-G. Several tissues from the laser group became too fragmented to image, leaving *n* = 7 for the microcrystal treated group and *n* = 12 for the sham group. For each retinal wholemount, 12 images were taken from the region 2–3 mm from the optic nerve (3 images per each of four retinal quadrants) at 40× magnification with Zeiss 710 Confocal Microscope(Carl Zeiss, White Plains, NY, USA). Confocal images were viewed in ImageJ (National Institute of Health) without modification of contrast or brightness. RGCs were identified by co-staining with DAPI and β-III tubulin. Investigators were masked as to the treatment when processing and imaging the tissues. RGCs were manually counted in a masked fashion by two independent observers. A third person was randomly assigned to count 9 out of the 12 images per retina flatmount to ensure consistency of counting results. If the count by the third observer was different than the average of the first two counters by more than ± 10%, the images were recounted by all three observers before being averaged. For each animal, the number of RGCs was normalized to the healthy contralateral eye and reported as a percentage.

### 2.13. Corneal Neovascularization Model

Sprague Dawley rats (6–8 weeks old) were used. Corneal neovascularization was induced by placing 10–0 nylon sutures superficially in the cornea, as previously reported with some modification [32]. Rats were anesthetized and given a topical proparacaine solution as a local anesthetic of the ocular surface. One drop of iodine solution was instilled to disinfect the ocular surface. Two stitches of Nylon suture were placed in the right eye 1 mm apart in the superior cornea, 2–3 mm from the limbus, using an operating microscope. The placed suture remained in the cornea until the end of the experiment. Immediately after suture implantation, rats received a unilateral anterior 5 µL subconjunctival injection of SPC microcrystals (100 µg sunitinib, 0.04% (*w*/*v*) PS80, 0.5% (*w*/*v*) methylcellulose) using a 27-gauge needle. After the procedures, antibiotic (neomycin, polymyxin, bacitracin) ointment was applied to prevent possible infection. After 7 days, the growth of blood vessels in the cornea was imaged using a Nikon SMZ stereoscope (Melville, NY, USA) at 10× magnification. The area of neovascularization area was quantified by drawing arc along the limbus and the neovascularization area. The area was measured in pixels and quantified by the dividing by the number of pixels in 1 mm^2^ using Adobe Photoshop.

### 2.14. Histology

Brown Norway rats (6–8 weeks old) were anesthetized and received unilateral subconjunctival injection of either SPC microcrystals (200 µg/5 µL sunitinib, 0.04% PS80, 0.5% methylcellulose) or vehicle (5 µL 0.04% PS80, 0.5% methylcellulose) using a 27-gauge needle. At 1 week and 20 weeks after injection, eyes were enucleated and placed in 4% paraformaldehyde prior to paraffin embedding, sectioning, and H&E staining by the Johns Hopkins Reference Histology Laboratory. Histological sections were evaluated in a masked manner by a board-certified ophthalmic pathologist for signs of inflammation and tissue damage.

### 2.15. Statistical Methods

Statistical analyses of two groups were conducted using a two-tailed Student’s *t*-test, two-way analysis of variance (ANOVA), or a two-tailed Mann–Whitney test. One-way ANOVA with Dunnett’s multiple comparison test was used for comparison of multiple groups. Statistical analysis was done using GraphPad Prism 9 (San Diego, CA, USA). Differences were considered to be statistically significant at a level of *p* < 0.05. For laser-induced ocular hypertension, cumulative IOP exposure was calculated using the trapezoidal rule. Outcome variables were considered to be normally distributed if −0.8 < skewness < 0.8 and −3.0 < kurtosis < 3.0. For comparisons of microcrystal rats to sham rats, unpaired Student’s *t*-test was used for normally distributed outcomes. For outcomes not normally distributed, the Wilcoxon rank sum test for two independent groups was used.

## 3. Results

### 3.1. Sunitinib Complex Microcrystal Formulation and Characterization

**Figure 1:**
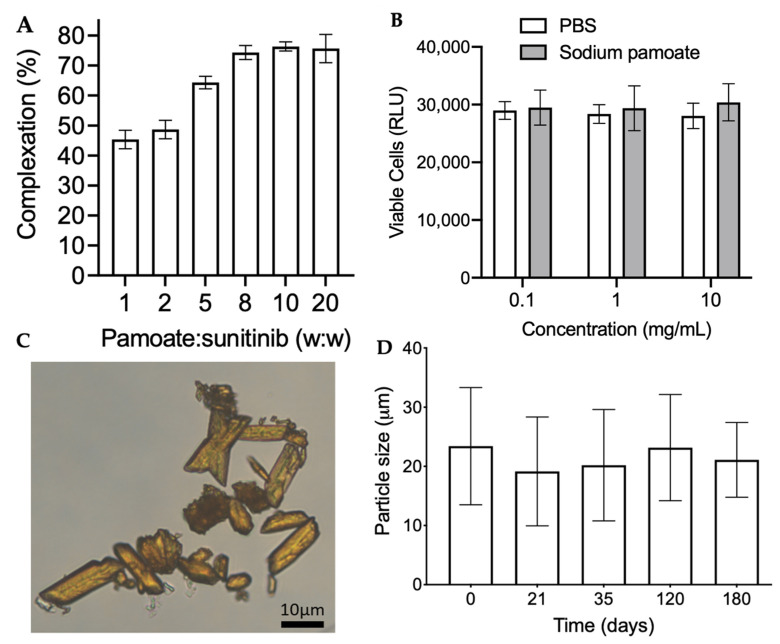
SPC microcrystal characterization. (**A**) Complexation efficiency of sunitinib malate when mixed at various ratios of pamoic acid to sunitinib (*n* = 3). Data shown as mean ± SD. (**B**) Viability of primary mouse RGCs after treatment with sunitinib malate (1 µM) plus either sodium pamoate or PBS (*n* = 3–5). Data represented as the mean ± SEM. (**C**) Representative microscopy image of SPC microcrystals. Scale bar = 20 μm. (**D**) Volume mean size (µm) of SPC microcrystals over a period of 180 days at room temperature (*n* = 3). Data shown as mean ± SD.

We first screened hydrophobic anionic salts as potential ion pairs for sunitinib malate. Linoleic acid sodium salt, sodium stearate, and sodium decanoate formed an oily substance when mixed 1:1 with sunitinib malate. In contrast, pamoic acid disodium formed a solid precipitate when mixed with sunitinib malate, and thus, was used for further formulation. It was found that a minimum of 1 h complexation time was required to maximize yield and room temperature was suitable (not shown). As shown in Figure 1sA, the complexation efficiency increased with increasing excess of pamoic acid, with a plateau around 8–10-fold excess of pamoic acid resulting in complexation of ~75% of the sunitinib malate in solution. Importantly, pamoic acid was found to have no detrimental effect on primary mouse RGC survival compared to PBS at concentrations as high as 10 mg/mL (Figure 1B). Using NMR, it was confirmed that the ratio of sunitinib to pamoate in the sunitinib-pamoate complex (SPC) was 1:1 (Figure S1). The solubility of the SPC in PBS at pH 7.4 was decreased ~34-fold compared to sunitinib malate, 15.3 ± 1.4 µg/mL vs. 525 ± 63 µg/mL, respectively.

Microcrystal formulation conditions were then screened, where the SPC complex was observed to have the highest solubility in ethanol/water (Figure S2). Microcrystals formed when using 60–70% ethanol as the solvent, whereas other mixtures either remained solutions or formed uncontrolled aggregates (Figure S3). The final SPC microcrystal formulation was confirmed to be crystalline by X-ray diffraction (Figure S4). The SPC microcrystals showed a rod-shaped morphology (Figure 1C), which when measured by image analysis showed approximate length of 25 ± 7 µm and width of 10 ± 2 µm. The drug loading as a percentage of the total mass was found to be 50.5 ± 2.9%, and the final yield was 37 ± 7.2%. Surprisingly, the SPC microcrystals showed relatively stable size as measured by the Coulter counter without additional surface stabilizers when stored at or above 5 mg/mL at room temperature for up to 180 days (23.4 ± 9.9 µm on day 0 vs. 21.1 ± 6.3 µm at 180 days, Figure 1D).

### 3.2. Ocular Pharmacokinetics and Efficacy in a Rat Model of Optic Nerve Crush with Subconjunctival Injection of SPC Microcrystals 

We next sought to investigate the effect of subconjunctival injection volume on ocular pharmacokinetics. As the microcrystals have higher drug loading than typical polymeric microparticles, we were able to inject similar doses to what we have investigated previously for preventing corneal neovascularization [25] with as little as 5 µL. We compared different SPC microcrystal doses to determine a dose that would result in therapeutically relevant sunitinib concentrations in the retina [28]. We found that the 200 µg dose provided significantly higher median retinal combined sunitinib and *N*-desethyl sunitinib drug concentrations that may be sufficient for protection of RGCs one week after dosing (Figure S5A). We then investigated the effect of volume when injecting the same sunitinib dose (200 µg), but in 5 µL compared to 100 µL volume in rats. One week after injection, the combined median drug concentration was ~18-fold higher in the retina for the 5 µL vs. 100 µL injection (Figure S5B). In rabbits, the combined median drug concentration was ~4.5-fold higher in the retina when injecting 200 µg of SPC microcrystals in 5 µL vs. 500 µL volume (Figure S5C).

**Figure 2:**
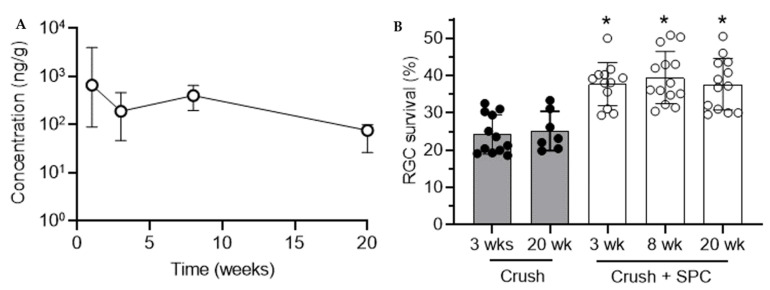
Pharmacokinetics and efficacy in protecting retinal ganglion cells (RGCs) in a rat model. (**A**) A single unilateral subconjunctival injection of SPC microcrystals (200 μg in 5 μL) was given in rats, and tissues were analyzed for combined sunitinib and *N*-desethyl sunitinib levels in the retina at the specified time points (*n* = 4–9). Units shown as ng combined drug per g of tissue (ng/g). Data shown as median ± IQR. (**B**) SPC microcrystals (200 μg in 5 μL) were injected unilaterally and an optic nerve crush procedure was performed 2 weeks prior to the indicated time points (*n* = 7–12). After 2 weeks, the percentage of surviving RGCs was calculated compared to the healthy fellow eye. Data shown as mean ± SD. * *p* < 0.05 compared to control crush animals at 3 and 20 weeks. Statistical analyses conducted by one-way ANOVA with multiple comparisons.

**Figure 3:**
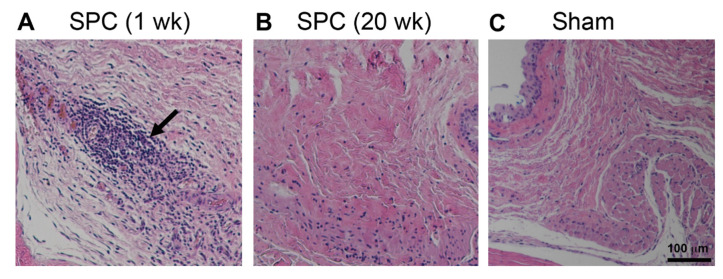
SPC microcrystals were well-tolerated in the subconjunctival space in rats. Either SPC microcrystals (200 μg in 5 μL) or vehicle (Sham) were injected unilaterally (*n* = 3). Representative hematoxylin and eosin-stained images of conjunctiva tissue sections showed (**A**) mild accumulation of inflammatory cells at the injection site at 1 week after SPC microcrystal injection, which (**B**) was largely resolved before 20 weeks, similar to (**C**) vehicle injection at 1 week. Scale bar = 100 μm and applies to all images.

We then assessed the longitudinal pharmacokinetics of the 200 µg sunitinib/5 µL subconjunctival SPC microcrystal dose in rats for up to 20 weeks. The combined drug levels were relatively stable, with a median value of 69.2 ng/g in the retina at 20 weeks (Figure 2A). Indeed, these drug levels provided by subconjunctival injection of SPC microcrystals were found to be protective of RGCs when performing optic nerve crush at time points up to 20 weeks. We ensured that the age of the rat at the time of the optic nerve crush procedure did not affect the RGC survival in control animals, as the RGC survival was indistinguishable at 3 weeks (24 ± 5.3%) and 20 weeks (25 ± 5.3%) (Figure 2B). In contrast to control animals, SPC microcrystal treated animals showed a significant increase in RGC survival at 3 weeks (38 ± 5.8%), 8 weeks (39 ± 7.0%) and 20 weeks (38 ± 6.9%) after microcrystal injection (Figure 2B). Representative images of the stained retinal flatmounts with arrows marking representative RGC cells co-stained with γ-synuclein and βIII tubulin are shown in Figure S6. Histological assessment of the tissue around the subconjunctival injection site in healthy rats showed an acute mild inflammatory reaction at 1 week that was resolved before 20 weeks after injection of SPC microcrystals (Figure 3). The retinas of rats receiving subconjunctival injection of SPC microcrystals showed no notable signs of toxicity compared to sham vehicle injection at 1 week (Figure S7).

### 3.3. SPC Microcrystal Efficacy in a Model of IOP Elevation 

**Figure 4:**
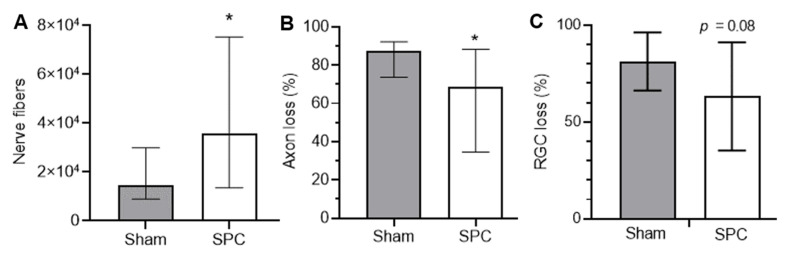
SPC microcrystals preserve nerve fibers and axons. A single unilateral subconjunctival injection of SPC microcrystals (200 μg in 5 μL) or vehicle (Sham) was prior to laser induced hypertension (*n* = 15–16). Optic nerves were evaluated for (**A**) number of nerve fibers and (**B**) percent reduction in nerve axons. Data shown as median ± IQR. * *p* < 0.05. Statistical analyses conducted by Wilcoxon rank sum test for two independent groups. (**C**) Retinal ganglion cell (RGC) loss was reduced with SPC microcrystals (*n* = 7) compared to untreated animals (*n* = 12), but not statistically significant (*p* = 0.08) due to variability and loss of tissues during the staining process. Data shown as mean ± SD. Statistical analysis conducted by unpaired *t*-test.

Next, we tested the SPC microcrystals in a rat model of glaucoma where RGC death and axonal damage is driven by induced IOP elevation, similarly to human disease, rather than mechanical injury. Model induction was consistent across the treated and control groups, as the cumulative IOP elevation over the 6 weeks after laser was not different between sham and SPC microcrystal treated eyes (Table S1). IOP elevation in this model is associated with increased axial length and width, which were not different between treated and sham eyes (Table S1). SPC microcrystal injected glaucoma eyes had significantly more axonal nerve fibers than sham injected glaucoma eyes (35,989 vs. 14,418, *p* = 0.04) (Figure 4A), and reduced axonal loss compared to sham injected glaucoma eyes (68.8% vs. 87.4%, *p* = 0.04) (Figure 4B). There was a trend toward a reduction in RGC loss with SPC microcrystals (63.3% ± 28% vs. 81.3% ± 15%, *p* = 0.08), though statistical significance was not reached due to the reduced number of tissue samples that remained intact throughout the staining process (Figure 4C).

### 3.4. Ocular Pharmacokinetics with Subconjunctival SPC Microcrystal Injection in Pigs

**Figure 5:**
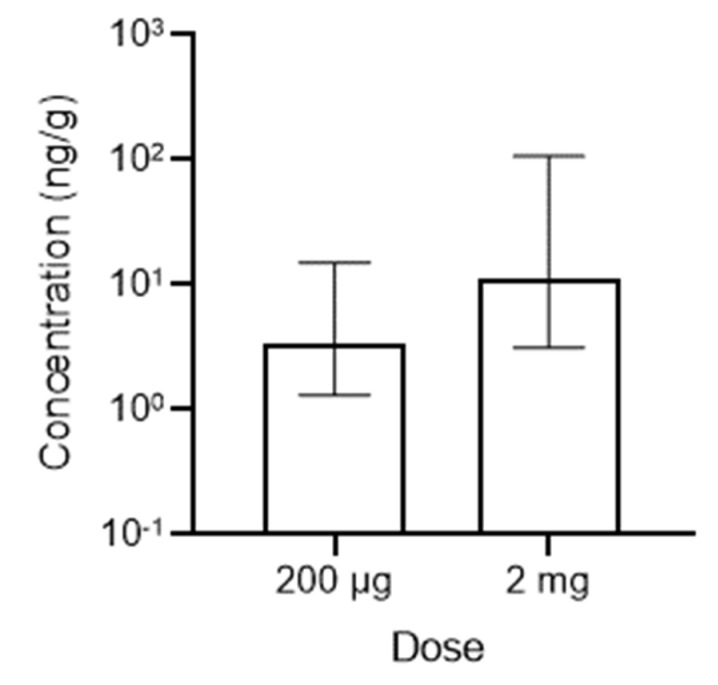
Therapeutically relevant drug concentrations were achieved in the retinas of pigs. A single unilateral subconjunctival injection of SPC microcrystals at a dose of either 200 μg (5 μL) or 2 mg (50 μL) was given in pigs, and tissues were analyzed for combined sunitinib and *N*-desethyl sunitinib levels in the retina at the specified time points (*n* = 4). Units shown as ng combined drug per g of tissue (ng/g). Data shown as median ± IQR.

We then sought to determine whether therapeutically relevant drug concentrations could be achieved with subconjunctival injection of SPC microcrystals in an animal with larger eyes and thicker sclera, such as pigs. Due to the larger size, we tested the 200 µg dose and a larger dose of 2 mg (Figure 5). The median combined retinal drug concentration observed in pigs at 7 days after injecting the 2 mg dose (11.1 ng/g) was not statistically different from the concentrations that maintained efficacy in protecting RGCs in rats at 20 weeks in this study (shown in Figure 2A), and similar to effective concentrations observed in a separate study using an eye drop formulation (~25.6 ng/g [28]). Similar to what was observed in the longitudinal study in rats (Figure S8A), combined drug levels in the choroid and retinal pigment epithelium (Ch/RPE) were ~10-fold higher than levels in the retina in pigs (Figure S8B), likely indicative of the known melanin binding properties of sunitinib [28].

### 3.5. Ocular Pharmacokinetics and Efficacy of Subconjunctival SPC Microcrystals in Preventing Corneal Neovascularization

**Figure 6:**
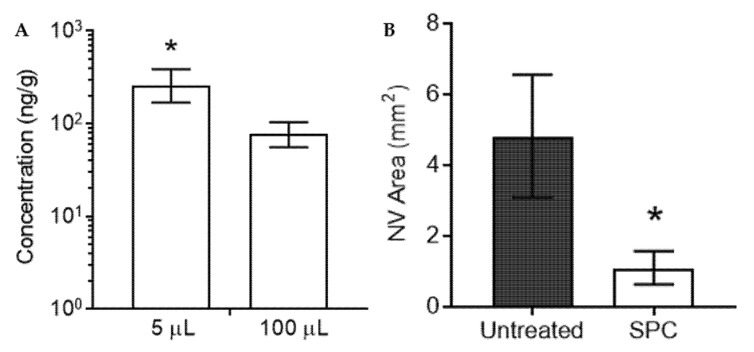
Subconjunctival SPC microcrystals suppress corneal neovascularization in a rat model. (**A**) A single unilateral subconjunctival injection of 100 μg SPC microcrystals was given to rats in two different injection volumes (5 and 100 μL). After 7 days, cornea tissues were collected and analyzed for combined levels of sunitinib and *N*-desethyl sunitinib (*n* = 4–6). Units shown as ng combined drug per g of tissue (ng/g). Data shown as median ± IQR. * *p* < 0.05 (**B**) SPC microcrystals (100 μg in 5 μL) were injected unilaterally at the same time as suturing the cornea to induce neovascularization. SPC microcrystals suppressed neovascularization for up to 1 week compared to untreated control animals (*n* = 4–8). Data shown as mean ± SD. * *p* < 0.05. Statistical analyses conducted by Student’s *t*-test.

Sunitinib was developed as a tyrosine kinase inhibitor, and thus, better known for its antiangiogenic properties via inhibition of vascular endothelial growth factor receptors (VEGFR) and platelet-derived growth factor receptors (PDGFR) [33]. We previously demonstrated that subconjunctival injection of polymeric microparticles to deliver sunitinib malate provided inhibition of corneal neovascularization in rats, whereas injection of free sunitinib malate in solution had no effect on neovascularization [25]. Based upon these results, we tested whether anterior subconjunctival injection of SPC microcrystals could inhibit corneal neovascularization. Similar to what was observed for the retina, subconjunctival injection of a 100 µg dose of SPC microcrystals in a 5 µL volume resulted in increased median drug concentrations in the cornea (321 ng/g) at 7 days after injection compared to a 100 µg dose injected in a 100 µL volume (96.3 ng/g) (Figure 6A). When 100 µg of SPC microcrystals was administered by subconjunctival injection in a 5 µL volume in a rat model of induced corneal neovascularization, the area of new blood vessel growth was reduced by 4.4-fold after 7 days compared to untreated rats (1.1 ± 0.2 mm^2^ vs. 4.8 ± 0.6 mm^2^, *p* < 0.05) (Figure 6B).

## 4. Discussion

While elevated IOP is an important risk factor implicated in the onset and progression of glaucoma, studies have shown that disease progression continues in some patients despite IOP lowering [34]. Thus, efforts are underway to develop molecules that promote the survival of RGCs independent of IOP, which may be used as a complementary therapy to standard IOP lowering regimens [34]. Many glaucoma patients are prescribed complex eye drop regimens that may require dosing multiple times per day as well as multiple types of drops, which leads to issues with adherence [13,35,36]. Thus, an injectable sustained-release strategy may be attractive for achieving long-lasting neuroprotection in glaucoma patients. Here, we demonstrated that a single subconjunctival injection of SPC microcrystals provided sustained neuroprotection in a rat optic nerve crush model for at least 20 weeks.

Among the various periocular injections, subconjunctival injection is considered as one of the safer ways to administer drugs because the tip of the needle is visible throughout the procedure, thereby minimizing the risk associated with hemorrhage or perforation of the eye [37,38,39]. Furthermore, subconjunctival injection of steroids was shown to provide higher intraocular bioavailability with lower systemic absorption when compared to peribulbar or oral administration [40,41]. It was previously shown that subconjunctival injection of water-soluble drugs leads to increased intraocular bioavailability, potentially due to the higher concentration gradient and increased transscleral drug penetration [41,42,43,44]. However, water-soluble drugs are cleared from the subconjunctival space within hours [25] limiting the duration of therapeutic effect that can be achieved. Thus, drug delivery formulations for subconjunctival injection would ideally encapsulate and provide sustained release of water-soluble drugs, but prior attempts at this approach have been difficult to achieve.

We previously described approaches for loading water soluble dexamethasone sodium phosphate and sunitinib malate into polymeric particles for subconjunctival injection, providing sustained prevention or treatment of corneal graft rejection [42], corneal neovascularization [25,45] and uveitis [24]. However, one potential drawback was relatively low drug loading in the range of 6–8% by weight. Here, a 1:1 molar ratio of pamoate and sunitinib in the SPC microcrystals was observed, which was consistent with the dicarboxylic acid structure of pamoic acid, similar to the conventional malic acid used in the water-soluble pharmaceutical salt. However, in contrast to the water-soluble malic acid salt, complexation with pamoic acid led to the formation of a poorly soluble precipitate that could be recrystallized into SPC microcrystals. Furthermore, we unexpectedly observed that the SPC microcrystals were stable over 180 days at room temperature at a concentration of at least 5 mg/mL without the addition of various stabilizing surfactants and polymers, such as PS80 and Pluronic polymers. The addition of these stabilizers either increased heterogeneity or led to dissolution of the SPC microcrystals. The combination of the 1:1 ratio and the lack of need for stabilizing polymer excipients resulted in ~50% drug loading in the SPC microcrystals. Higher drug loading allows for smaller injection volumes, which here resulted in increased drug delivery to the posterior segment (injection placed more posteriorly) and to the cornea (injection placed more anteriorly). It is possible that the lower volume and lower surface area for drug absorption led to decreased drug clearance via conjunctival blood vessels and lymphatic drainage [46,47]. Smaller injection volumes may also be attractive for cosmetic reasons and may reduce the potential for tissue fibrosis with repeated injections over the duration of glaucoma treatment, which for the average patient lasts 13–16 years [48].

As with other modes of ocular administration, the choice of animal model is important when evaluating drug delivery to the posterior segment with subconjunctival injection. Mice and rats are often useful in terms of availability of disease models that can be used to demonstrate therapeutic efficacy and determine the range of drug concentrations that are effective. However, to more accurately predict whether drug could be delivered at therapeutically relevant concentrations in human eyes, larger animals must often be used. Compared to rodents and rabbits, pigs have ocular structural features that may more closely recapitulate the human eye. For example, the pig scleral thickness range of 0.3–0.8 mm is similar to the range of thickness reported for humans (0.4–0.9 mm), as is the choroidal blood flow rate (~1.6 mL/min for pigs and 1.4 mL/min for humans) [49,50,51,52]. In contrast, scleral thickness in mice and rats is much thinner, 0.04 mm and <0.1 mm, respectively [31,53]. Thus, it is not surprising that lower drug levels in the retina were observed when injecting the same dose of SPC microcrystals in pigs compared to rats. A ten-fold higher dose in pigs resulted in retinal drug concentrations that reached the range of that was observed to be effective in protecting RGCs in rats [28]. The minimum effective concentration of sunitinib for protecting RGCs is not known, though future studies could include further dose ranging studies to look for minimum effective levels in models of RGC loss. Next steps in development of the SPC microcrystal formulation could also include additional dose ranging and longitudinal pharmacokinetic studies in pigs to achieve therapeutic drug levels for several months.

Another factor that can affect drug pharmacokinetics and pharmacodynamics is the binding of drugs to ocular melanin. It has been demonstrated that drug binding to melanin in the sclera can reduce the amount that is able to reach the posterior segment after subconjunctival injection [54,55,56]. In another study using an eye drop formulation, we demonstrated that binding of sunitinib to melanin in the choroid and retinal pigment epithelium (Ch/RPE) appeared to be beneficial for increasing the amount drug that reached the retina [28]. Similarly, we observed here that the drug levels in the Ch/RPE in pigmented rats and pigs was at least 10-fold higher than drug concentrations in the retina. Thus, for drugs that naturally bind to melanin, such as sunitinib, the use of pigmented animals is important. However, this can be an issue when disease models require the use of albino animals, such as was the case with the induced ocular hypertension model described here [57]. In this model, the laser-induced photocoagulation of the trabecular meshwork is negatively impacted by increased pigmentation in the trabecular meshwork, which limits the potential for induced IOP elevation [57,58]. With subconjunctival injection in albino animals, the benefit of reduced binding of drug to melanin in the sclera may be offset by the reduced binding in the choroid/RPE, leading to less effective delivery to the RGCs. In contrast, the optic nerve crush model used here is a mechanical model of RGC injury that has similar effects in albino and pigmented rats [28]. In pigmented rats, a single subconjunctival injection of 200 µg SPC microcrystals provided sustained retina drug concentrations and protection of RGCs for at least 20 weeks.

Many FDA-approved drugs have moderate to high water solubility through ionization and formulation as a pharmaceutical salt to enhance biodistribution [43,59]. The approach described here for ionic complexation to reversibly lower the water solubility for formulation for sustained release could be applied to numerous other pharmaceuticals. The SPC microcrystal formulation has high translational potential, as oral sunitinib malate (Sutent^®^) is FDA-approved for treatment of some cancers. Furthermore, an intravitreal polymeric microparticle formulation that provides sustained intraocular delivery of sunitinib malate is in Phase 2 clinical trials for neovascular age-related macular degeneration [60] and macular edema (ClinicalTrials.gov Identifier: NCT04085341). Pamoic acid has a history of safety as a counterion used in several oral drug formulations, though thorough safety studies will be required to demonstrate safety with subconjunctival injection. A safe and efficacious injectable formulation with the potential for twice-yearly administration for neuroprotection in glaucoma would likely have a significant impact in helping to maintain vision and quality of life.

## 5. Conclusions

Neuroprotection is a promising strategy to complement intraocular pressure (IOP) lowering for preserving retinal ganglion cells (RGCs) survival in glaucoma. Sunitinib malate is a water-soluble broad-spectrum protein kinase inhibitor with activity against dual leucine kinase (DLK) and leucine zipper kinase (LZK), which has been shown to prevent RGC death. Here, we describe an approach for formulating water insoluble ion-complex microcrystals containing ~50% sunitinib by mass. A single subconjunctival injection of sunitinib-pamoate complex (SPC) microcrystals provided 20 weeks of sustained retina drug concentrations and RGC protection in a rat model of optic nerve injury. Consistent with sunitinib’s ability to inhibit blood vessel growth, subconjunctival injection of SPC microcrystals also inhibited vessel growth in a rat model of corneal neovascularization. Importantly, therapeutically relevant retina drug concentrations were achieved with subconjunctival injection of SPC microcrystals in pigs. An injectable therapy that provides sustained RGC protection has the potential to improve the management of glaucoma.

## 6. Patents

H.T.H., Y.C.K., L.M.E., and J.H. are inventors on patents/patent applications related to this technology. D.J.Z., H.A.Q., and C.A.B. are inventors on patents related to sunitinib and neuroprotection.

## Data Availability

The main data supporting the findings of this study are available within the paper and its Supplementary information. The associated raw data are available from the corresponding author on reasonable request.

## Supplementary Materials

The following are available online at https://www.mdpi.com/article/10.3390/pharmaceutics13050647/s1, Table S1. Laser hypertension study outcomes for glaucoma eyes compared to contralateral control eyes. Figure S1. Sunitinib forms an insoluble complex with pamoic acid (sunitinib-pamoate complex, SPC); Figure S2. Ethanol/water mixtures yield the highest solubility; Figure S3. Anti-solvent optimization leads to uniform crystallization; Figure S4. SPC microcrystals are crystalline; Figure S5. Increasing SPC microcrystal dose and concentration (decreasing volume of injection (injection) results in increased retina drug concentrations; Figure S6. Representative images of stained retinal flatmounts used for RGCs counting; Figure S7. Intraocular sunitinib levels showed no sign of retinal toxicity at 1 week; Figure S8. Drug concentrations in the choroid/retinal pigment epithelium were ~10 fold higher than retina concentrations.
