# Supplementary material for: Ion-Complex Microcrystal Formulation Provides Sustained Delivery of a Multimodal Kinase Inhibitor from the Subconjunctival Space for Protection of Retinal Ganglion Cells
Source: Pharmaceutics. 2021 May 1;13(5):647. doi: 10.3390/pharmaceutics13050647 (PMC8147274; doi:10.3390/pharmaceutics13050647)
Supplement: Supplementary file 1 [file pharmaceutics-13-00647-s001.zip › pharmaceutics-1190496-supplementary.pdf]

pharmaceutics

Supplementary Materials: Ion-Complex Microcrystal Formulation Provides Sustained Delivery of a Multimodal Kinase Inhibitor from the Subconjunctival Space for Protection of Retinal Ganglion Cells

Henry T. Hsueh, Yoo-Chun Kim, Ian Pitha, Matthew D. Shin, Cynthia A. Berlinicke, Renee Ti Chou, Elizabeth Kimball, Julie Schaub, Sarah Quillen, Kirby T. Leo, Hyounkoo Han, Amy Xiao, Youngwook Kim, Matthew Appell, Usha Rai, HyeYoung Kwon, Patricia Kolodziejski, Laolu Ogunnaike, Nicole M. Anders, Avelina Hemingway, Joan L. Jefferys, Abhijit A. Date, Charles Eberhart, Thomas V. Johnson, Harry A. Quigley, Donald J. Zack, Justin Hanes and Laura M. Ensign

Table S1. Laser hypertension study outcomes for glaucoma eyes compared to contralateral control eyes. P values shown for comparison between glaucoma eyes from rats treated with SPC microcrystals (SPC) or vehicle (Sham). Values in shaded rows are shown in Figure 4. Student's t-test was used for normally distributed outcomes, and the Wilcoxon rank sum test for two independent groups was used for outcomes not normally distributed.

Outcome	Group (n)	Mean (SD) or Median (Q1–Q3)	*p*-Value
Cumulative IOP (mmHg × day)	SPC (16)	603.5 (101.3)	0.69
	Sham (15)	619.4 (117.0)	
Axial length (mm)	SPC (16)	6.76 (0.31)	0.21
	Sham (15)	6.63 (0.28)	
Axial width (mm)	SPC (16)	6.70 (0.16)	0.31
	Sham (15)	6.63 (0.21)	
Nerve density (fibers/mm^2)	SPC (16)	168,980 (68,720–334,340)	0.10
	Sham (15)	86,840 (47,240–181,000)	
Nerve area (mm^2)	SPC (16)	0.21 (0.02)	0.08
	Sham (15)	0.19 (0.03)	
Nerve fibers	SPC (16)	35,989 (13,559–75,378)	0.04
	Sham (15)	14,418 (8,899–29,921)	
Nerve diameter (mm)	SPC (16)	0.32 (0.06)	0.39
	Sham (15)	0.30 (0.05)	
Axon loss (%)	SPC (16)	68.8 (34.6–88.3)	0.04
	Sham (15)	87.4 (73.7–92.2)	
RGC loss (%)	SPC (7)	63.3 (28.0)	0.08
	Sham (12)	81.3 (15.0)	

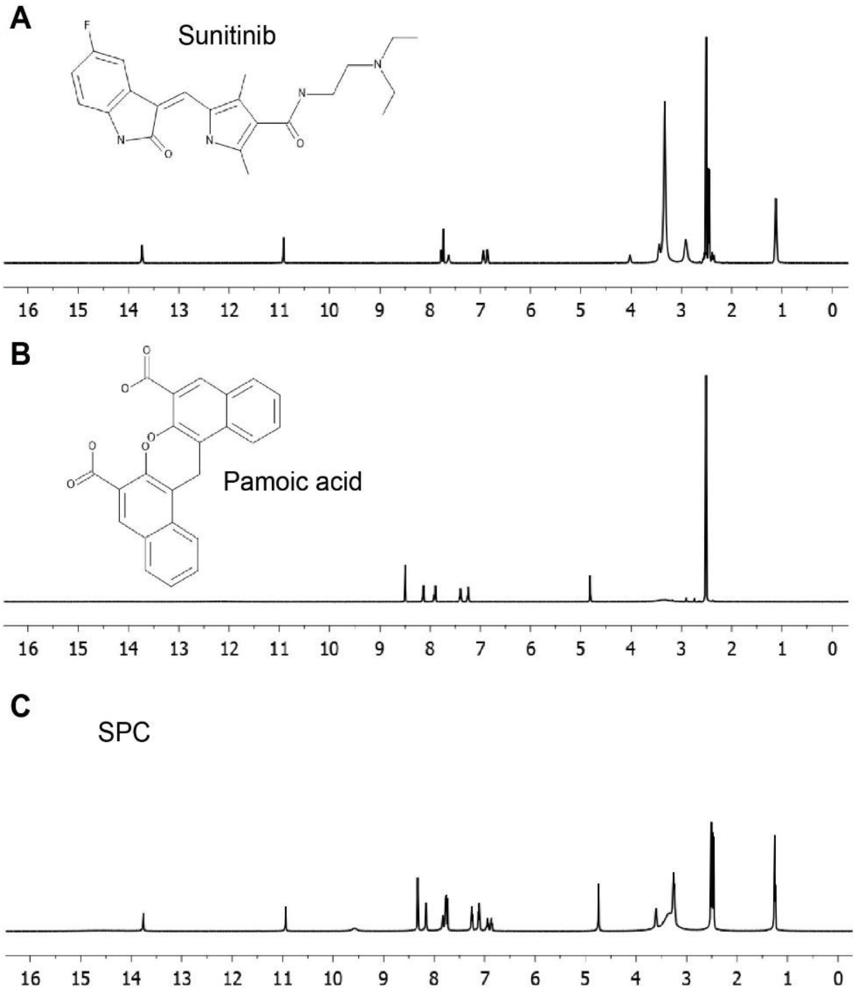

Figure S1. Sunitinib forms an insoluble complex with pamoic acid (sunitinib-pamoate complex, SPC). ^{1}H-NMR spectra of (A) sunitinib malate, (B) pamoic acid, and (C) sunitinib-pamoate complex (SPC).

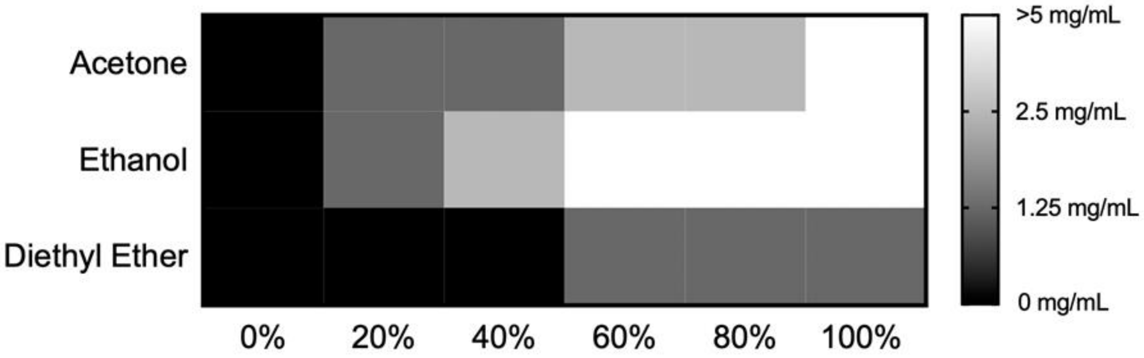

Figure S2. Ethanol/water mixtures yield the highest solubility. Chart showing solubility of the sunitinib-pamoate complex (SPC) in various organic solvents mixed with water at the volume ratios shown on the x-axis. SPC solubility was highest in ethanol/water mixtures.

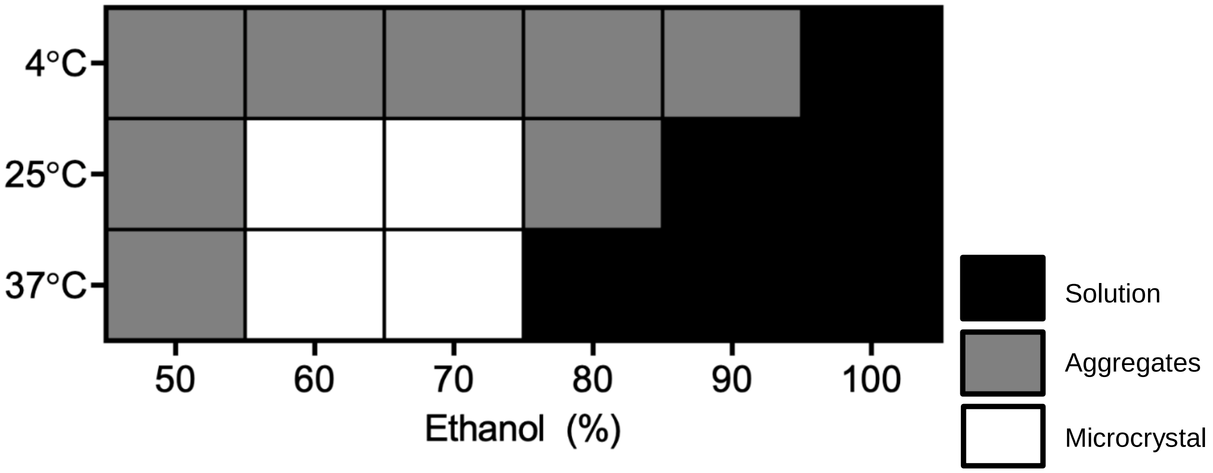

Figure S3. Anti-solvent optimization leads to uniform crystallization. Chart showing the outcome of stirring solutions of SPC dissolved in ethanol/water solutions at various temperatures (refrigeration, 4°C; room temperature, 25°C; water bath, 37°C) for 2 h. Outcomes included remaining a solution, forming aggregates that adhered to the container wall and stir bar, or forming microcrystals.

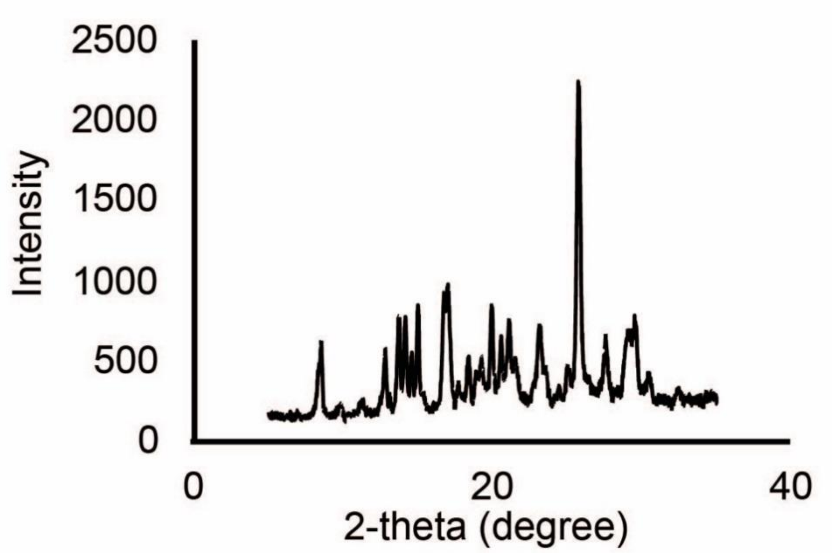

Figure S4. SPC microcrystals are crystalline. Powder X-ray diffraction (XRD) pattern of sunitinib-pamoate complex (SPC) microcrystals.

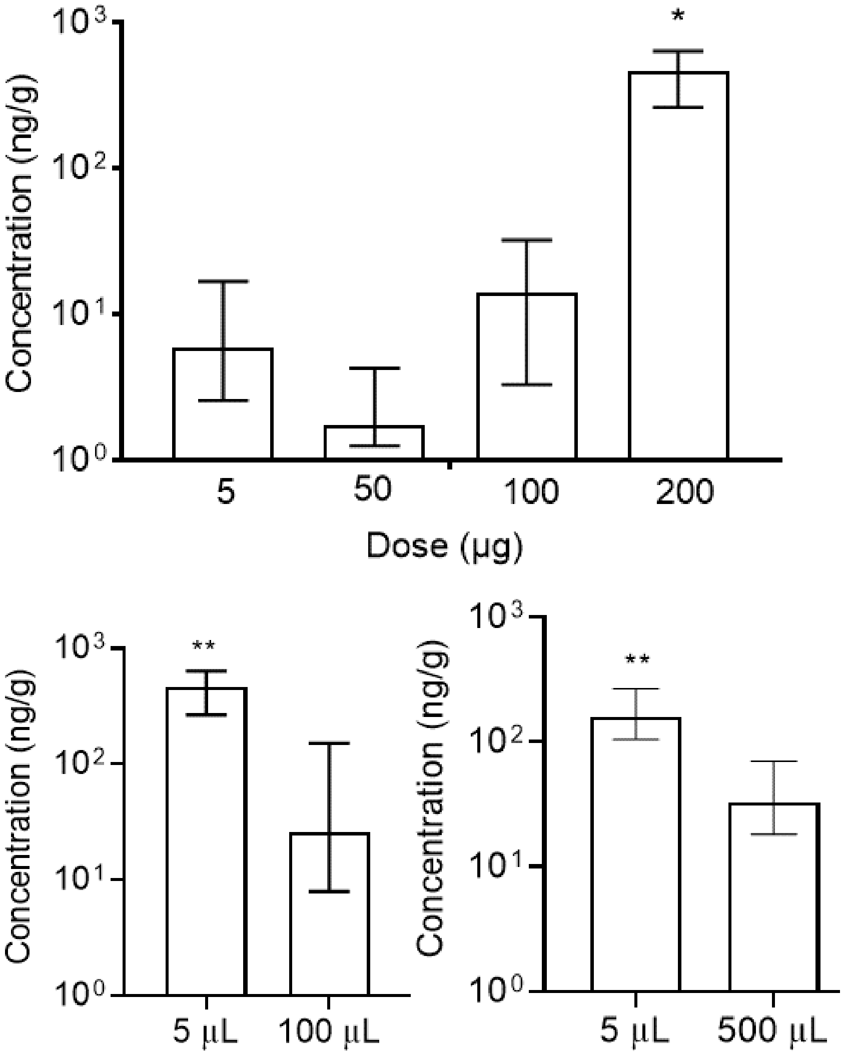

Figure S5. Increasing SPC microcrystal dose and concentration (decreasing volume of injection (injection) results in increased retina drug concentrations. (A) A single unilateral subconjunctival injection of different doses of SPC microcrystals in 5 L volume was given to rats. After 7 days, retina tissues were collected and analyzed for combined levels of sunitinib and N-desethyl sunitinib (n = 5-6). Units shown as ng combined drug per g of tissue (ng/g). Data shown as median ± IQR. *$p < 0.05$ compared to all other doses. (B) A single unilateral subconjunctival injection of 200 g SPC microcrystals was given to rats in two different injection volumes (5 and 100 L). After 7 days, retina tissues were collected and analyzed for combined levels of sunitinib and N-desethyl sunitinib (n = 5). Units shown as ng combined drug per g of tissue (ng/g). Data shown as median ± IQR. **$p < 0.01$. (C) A single unilateral subconjunctival injection of 200 g SPC microcrystals was given to rabbits in two different injection volumes (5 and 500 L). After 7 days, retina tissues were collected and analyzed for combined levels of sunitinib and N-desethyl sunitinib (n = 4-5). Units shown as ng combined drug per g of tissue (ng/g). Data shown as median ± IQR. **$p < 0.01$.

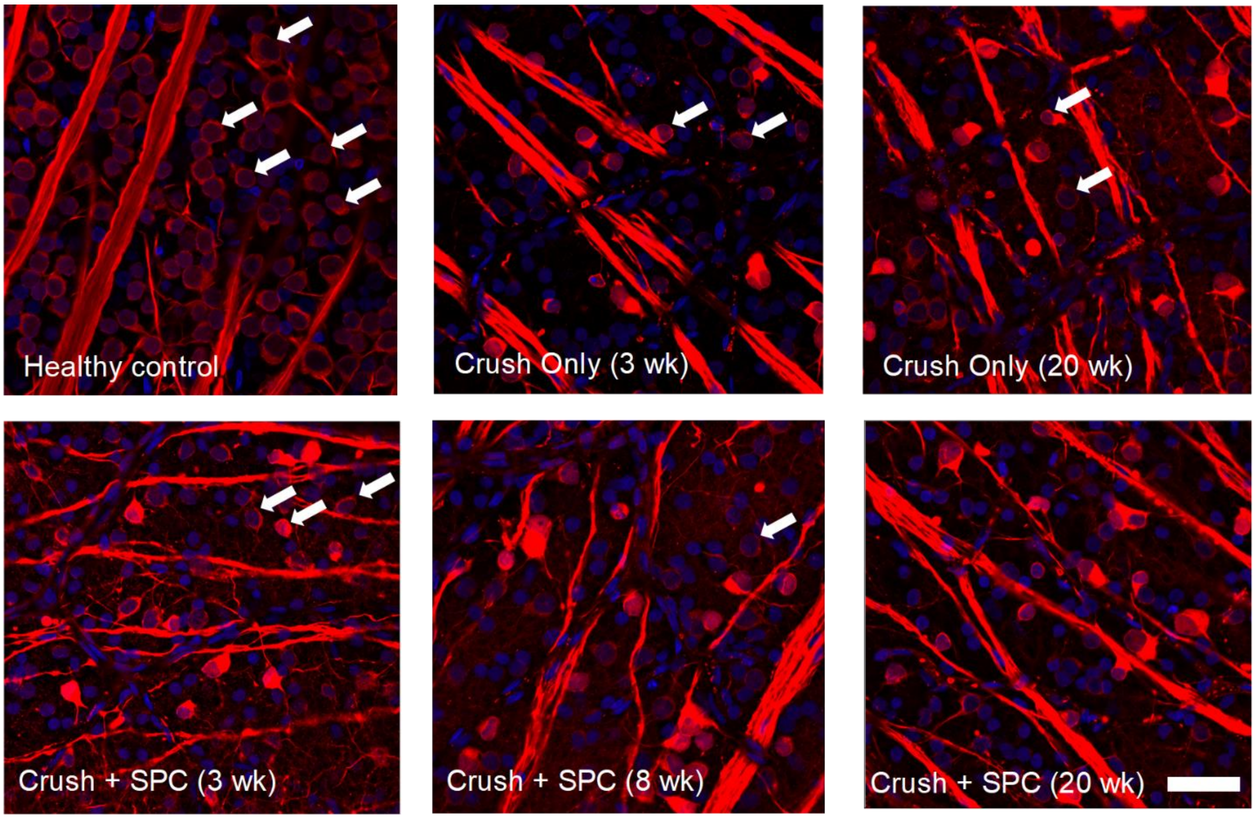

Figure S6. Representative images of stained retinal flatmounts used for RGC counting. SPC microcrystals (200 μg in 5 μL) were injected unilaterally and an optic nerve crush procedure was performed 2 weeks prior to the indicated time points (n = 7–12). After 2 weeks, the percentage of surviving RGCs was calculated compared to the healthy fellow eye (healthy control). Representative images of retina tissue sections stained for γ-synuclein and βIII tubulin for (A) healthy control, optic nerve crush with vehicle at (B) 3 weeks and (C) at 20 weeks, and optic nerve crush + SPC microcrystals at (D) 3 weeks, (E) 8 weeks, and (F) 20 weeks. White arrows show cells co-stained with both markers, characteristic of RGCs. Scale bar = 50 m and applies to all images.

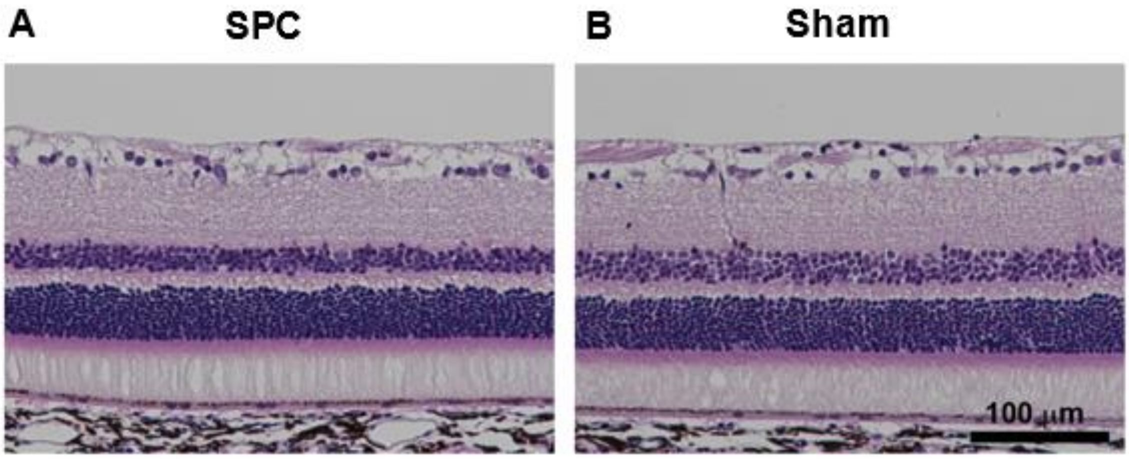

Figure S7. Intraocular sunitinib levels showed no sign of retinal toxicity at 1 week. A single unilateral subconjunctival injection of either SPC microcrystals (200 μg in 5 μL) or vehicle (Sham) was given to healthy rats (n = 3). Representative hematoxylin and eosin stained images of retina tissue sections showed no difference between (A) SPC microcrystals and (B) Sham at 1 week. Scale bar = 100 μm.

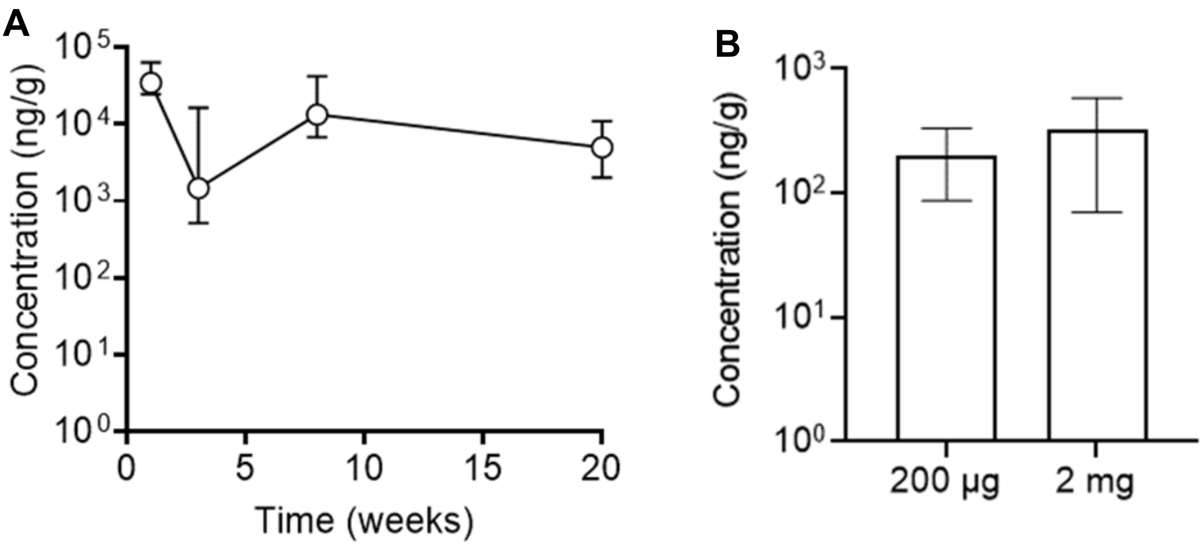

Figure S8. Drug concentrations in the choroid/retinal pigment epithelium were ~10 fold higher than retina concentrations. (A) A single unilateral subconjunctival injection of SPC microcrystals (200 g in 5 L) was given in rats, and tissues were analyzed for combined sunitinib and N-desethyl sunitinib levels in the choroid/RPE at the specified time points (n = 4 - 9). Data shown as median ± IQR. (B) A single unilateral subconjunctival injection of SPC microcrystals at a dose of either 200 g (5 L) or 2 mg (50 L) was given in pigs, and tissues were analyzed for combined sunitinib and N-desethyl sunitinib levels in the choroid/RPE at 1 week (n = 4). Data shown as median ± IQR. .
